# Observational evidence of herbivore‐specific associational effects between neighboring conspecifics in natural, dimorphic populations of *Datura wrightii*

**DOI:** 10.1002/ece3.7454

**Published:** 2021-03-26

**Authors:** Jay K. Goldberg, Sonya R. Sternlieb, Genevieve Pintel, Lynda F. Delph

**Affiliations:** ^1^ Department of Biology Indiana University Bloomington IN USA; ^2^ Wesleyan University Middletown CT USA; ^3^ University of Guelph Guelph ON USA; ^4^ Department of Ecology and Evolutionary Biology University of Arizona Tucson AZ USA

**Keywords:** associational effects, glandular trichomes, plant–herbivore interactions, stable polymorphism

## Abstract

Associational effects—in which the vulnerability of a plant to herbivores is influenced by its neighbors—have been widely implicated in mediating plant–herbivore interactions. Studies of associational effects typically focus on interspecific interactions or pest–crop dynamics. However, associational effects may also be important for species with intraspecific variation in defensive traits. In this study, we observed hundreds of *Datura wrightii*—which exhibits dimorphism in its trichome phenotype—from over 30 dimorphic populations across California. Our aim was to determine whether a relationship existed between the trichome phenotype of neighboring conspecifics and the likelihood of being damaged by four species of herbivorous insects. We visited plants at three timepoints to assess how these effects vary both within and between growing seasons. We hypothesized that the pattern of associational effects would provide rare morphs (i.e., focal plants that are a different morph than their neighbors) with an advantage in the form of reduced herbivory, thereby contributing to the negative frequency‐dependent selection previously documented in this system. We found the best predictor of herbivory/herbivore presence on focal plants was the phenotype of the focal plant. However, we also found some important neighborhood effects. The total number of plants near a focal individual predicted the likelihood and/or magnitude of herbivory by *Tupiochoris notatus*, *Lema daturaphila*, and *Manduca sexta*. We also found that velvety focal plants with primarily sticky neighbors are more susceptible to infestation by *Tupiochoris notatus* and *Lema daturaphila*. This does not align with the hypothesis that associational effects at the near‐neighbor scale contribute to a rare‐morph advantage in this system. Overall, the results of our study show that the number and trichome‐morph composition of neighboring conspecifics impact interactions between *D. wrightii* and insect herbivores.

## Graphical abstract

In this article, we demonstrate that intraspecific associational effects exist between neighboring plants within naturally dimorphic population of *Datura wrightii*. We use a generalized linear modeling approach to tease apart the relationship between the number and trichome phenotype of neighboring plants with the likelihood of herbivory present on focal plants. We find that focal plant trichome phenotype (glandular or nonglandular) was the main determinant of susceptibility differences, but also evidence for seasonal associational effects with respect to two insect herbivores: *Tupiochoris notatus* and *Lema daturaphila*.

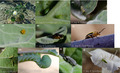

## 1 INTRODUCTION

Plant–plant interactions have attracted the attention of plant scientists since the first suggestion that plants can communicate with one another nearly 40 years ago (Baldwin et al., 2006; Baldwin & Schultz, 1983). Both positive and negative direct interactions between plants, occurring through mechanisms such as allelopathy or volatile‐mediated communication, have been described (Kalske et al., 2019; Latif et al., 2017). Indirect interactions between neighboring plants can also occur (Barbosa et al., 2009) and may be mediated by third parties in the environment such as herbivores, parasites, or natural enemies of herbivores (Barbosa et al., 2009). Associational effects are a category of plant–plant interactions that has received much attention from plant biologists in recent years (Barbosa et al., 2009; Underwood et al., 2014). Associational effects encompass all interactions in which neighboring plants alter the likelihood that a focal plant will be attacked by an herbivore and include effects that can be positive (associational resistance) or negative (associational susceptibility) from the perspective of a focal plant. Although associational effects are often studied in multispecies community contexts (Hambäck et al., 2014; Hay, 1986), they can also occur between conspecifics within polymorphic populations (Sato & Kudoh, 2016).

While multiple factors can contribute to the strength and direction of associational effects in plant populations and communities, herbivore population dynamics are particularly influential. When herbivore populations outgrow the supply of their preferred host plants, “spillover” onto less preferred hosts can occur, producing associational susceptibility (White & Whitham, 2000). Plant apparency has also been shown to drive associational effects, as smaller plants hidden among larger neighbors have been shown to experience associational resistance (Castagneyrol et al., 2013). Associational resistance can also occur when plants induce the production of volatile compounds, which can deter herbivores from ovipositing on the induced plant and its neighbors (Zakir et al., 2013). In addition to these biotic mechanisms, abiotic factors such as microclimate variation caused by neighbors can also drive associational effects (Barbosa et al., 2009).

*Datura wrightii* (Solanaceae) is a system that allows for the study of interactions between conspecifics in populations that are dimorphic with respect to trichome type. This roadside weed is common in California, where two distinct trichome morphs—glandular/sticky and nonglandular/velvety—coexist in many populations (Hare & Elle, 2001). These two morphs are easy to categorize by sight and touch in the field. Previous research using a statewide sample of populations has shown that this dimorphism is maintained by negative frequency‐dependent selection linked to two herbivorous insects (Goldberg et al., 2020). These two morphs do not produce significantly different herbivore‐induced volatile blends (Hare, 2007), but these blends do vary based upon the herbivore species (Hare & Sun, 2011). Here, we focus on intrapopulation plant–herbivore dynamics in this system. Experimental evidence from a study of dimorphic *Arabidopsis halleri* revealed that associational effects occurred between glabrous and hairy individuals (Sato & Kudoh, 2016). In this system, glabrous plants lacking trichomes were more palatable to herbivores (Sato et al., 2014) and hairy plants experienced associational resistance when surrounded by glabrous ones (Sato & Kudoh, 2016). We therefore hypothesize that associational effects may exist in dimorphic populations of *D. wrightii*.

In this paper, we present the results of an observational study of natural *D. wrightii* plants across central and southern California. Our goal was to assess the degree to which the number and trichome phenotype of neighboring conspecifics alters the likelihood of herbivory on dimorphic *D. wrightii*. Based upon prior work, we hypothesized that we would observe associational susceptibility (negative interactions from the perspective of a focal plant) occurring between neighboring plants with the same trichome phenotype and associational resistance (positive interactions) between neighbors with different trichome phenotypes. A scenario such as this could underlie the fitness cost associated with becoming common that occurs under negative frequency‐dependent selection as it would provide locally rare morphs (e.g., those that are different from their surrounding neighbors) a fitness advantage in the form of reduced herbivory compared to the locally common morph (e.g., those that are the same morph as their neighbors).

To test this hypothesis, we measured herbivory on California *D. wrightii* and counted the number and phenotype of neighboring plants to look for correlations between herbivory and neighborhood size/composition (local morph frequency). We conducted field measurements three times across two years (July/August 2017; April/May 2018; July/August 2018) so that in addition to testing for the presence of associational effects, we could also assess the degree to which it varied between and within growing seasons.

## 2 METHODS

### 2.1 Study system

*Datura wrightii* is a Solanaceous perennial shrub native to the American Southwest and Northwest Mexico. In California, these plants are dimorphic with respect to trichome type: Some plants possess nonglandular trichomes, whereas others possess glandular trichomes and feel sticky (Hare & Elle, 2001); we will refer to these as velvety and sticky, respectively. These phenotypes coexist within populations across the coastal regions of the state and differ with respect to their associated arthropod communities (Hare & Elle, 2002). Sticky plants have compromised indirect defenses (Gassmann & Hare, 2005), with the glandular phenotype conferring resistance to flea beetles (*Epitrix* sp.), vulnerability to mirid suckflies (*Tupiochoris notatus*), and proving less attractive to predatory arthropods. The trichome dimorphism is known to be governed by a single locus with classical Mendelian inheritance and is ontogenically expressed: All seedlings have glandular trichomes with the adult phenotypes exhibited following the emergence of the 5th true leaf (van Dam et al., 1999). Vegetative tissues senesce at the conclusion of the growing season and re‐emerge as their adult phenotype in each following year (Elle et al., 1999).

**FIGURE 1:**
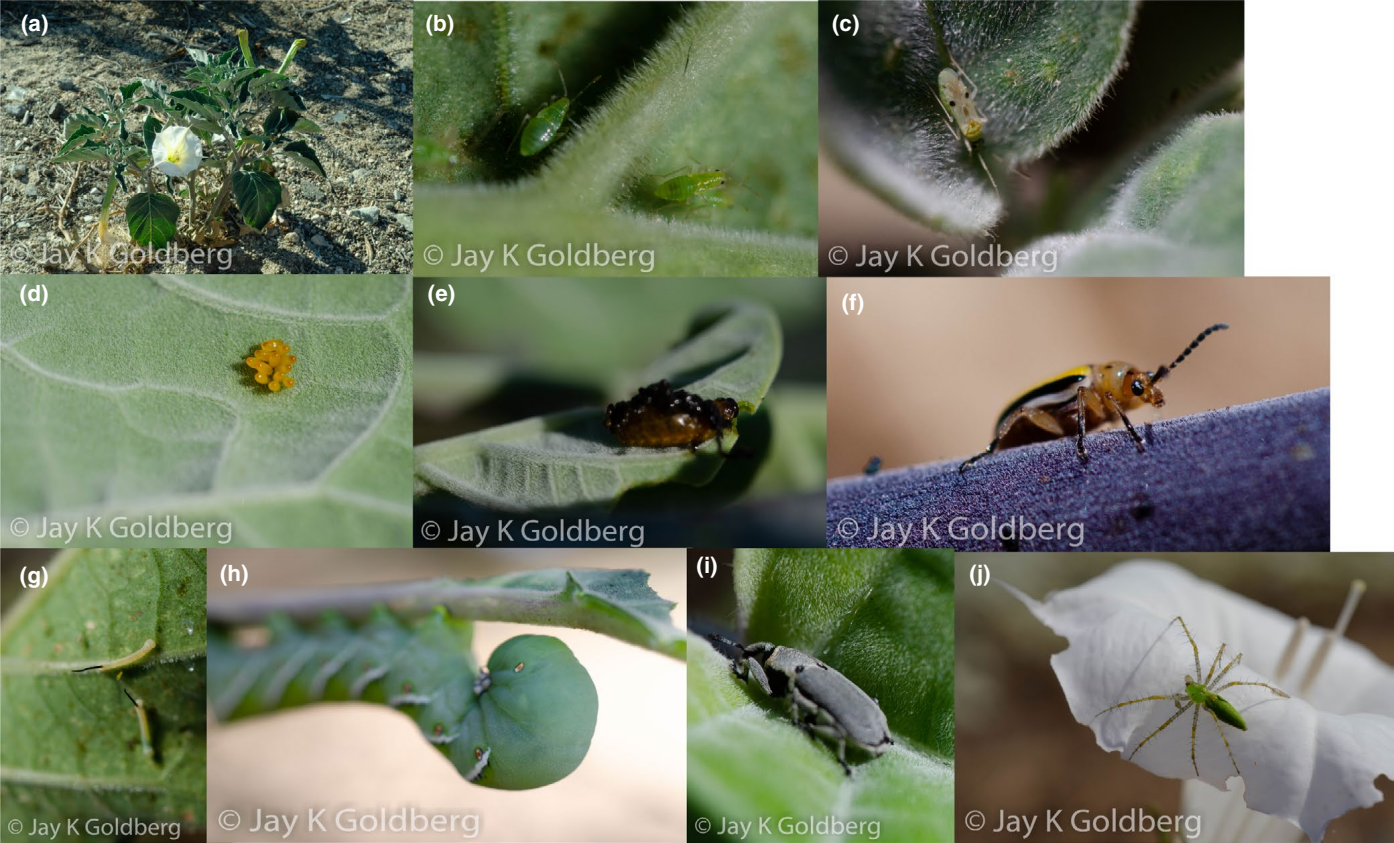
Photographs taken by JKG of study subjects in situ: *Datura wrightii* (a), *Tupiochoris notatus* (b nymphs; c adult), *Lema daturaphila* (d egg cluster; e larva; f adult), *Manduca sexta* (g early instar; h late instar), *Trichobaris compacta* (i adult), and Green Lynx Spider (*Peucetia viridans*; j). Other study species not shown are *Epitrix* sp. (flea beetles), predatory hemipterans (Geocoris sp., Berytidae), and uncommon spiders (Salticidae, Thomisidae, and Araneidae)

Our study focused on interactions between *D. wrightii* (Figure 1a) and five different species of herbivorous insect: *Manduca sexta, Lema daturaphila*, *Tupiochorus notatus*, *Epitrix* sp., and *Tricobaris compacta*. All these herbivores except *Epitrix* are shown in Figure 1 (panels b–i). *Manduca sexta* (Lepidoptera: Sphingidae) adults are not herbivorous but will oviposit on their solanaceous host plants—including *D. wrightii*. Their larvae are voracious eaters and are capable of defoliating entire plants as the caterpillars grow from less than 1 cm to over 10 cm over the course of 2–3 weeks (Reinecke et al., 1980). *Lema daturaphila* (Coleoptera: Chrysomelidae) is herbivorous during both larval and adult stages and oviposit on their host plants (Kogan & Goeden, 1970). Both *M. sexta* and *L. daturaphila* are chewing insects that completely remove vegetative tissues; however, *M. sexta* defoliates in a regular pattern consuming all parts of the leaf; *L. daturaphila* on the other hand leaves irregularly shaped damage and avoids eating veins (Hare & Elle, 2002). *Epitrix* flea beetles (Coleoptera; Chrysomelidae) are herbivorous as both adults and larvae; however, the eggs are laid in the soil at the base of host plants and the larvae feed on root tissue (Westdal & Romanow, 1972). As such, we only assessed damage by adults, which leave numerous small puncture wounds in leaves (Hare & Elle, 2002). *Tupiochorus notatus* (Hemiptera: Miridae) are small piercing‐sucking herbivores (as both nymphs and adults), the damage of which manifests as a distinctive yellow discoloration of leaves (Hare & Elle, 2002). *Trichobaris compacta* (Coleoptera: Curculionidae) adults leave small holes that are notably larger than those left by flea beetles. The larvae of this species are pith dwellers (Lee et al., 2016); thus, we did not determine their presence as it would require damaging plants and significantly disturbing their arthropod communities. Adults of all herbivore species are capable of dispersing via flight, whereas nymphs/larvae are flightless (J Goldberg, personal observation). The striking differences between the damage patterns of each herbivore allowed us to determine how much leaf area was damaged by each species individually for all focal plants included in this study. Detailed descriptions of these patterns of damage have been previously published (Elle & Hare, 2000).

### 2.2 Data collection

**FIGURE 2:**
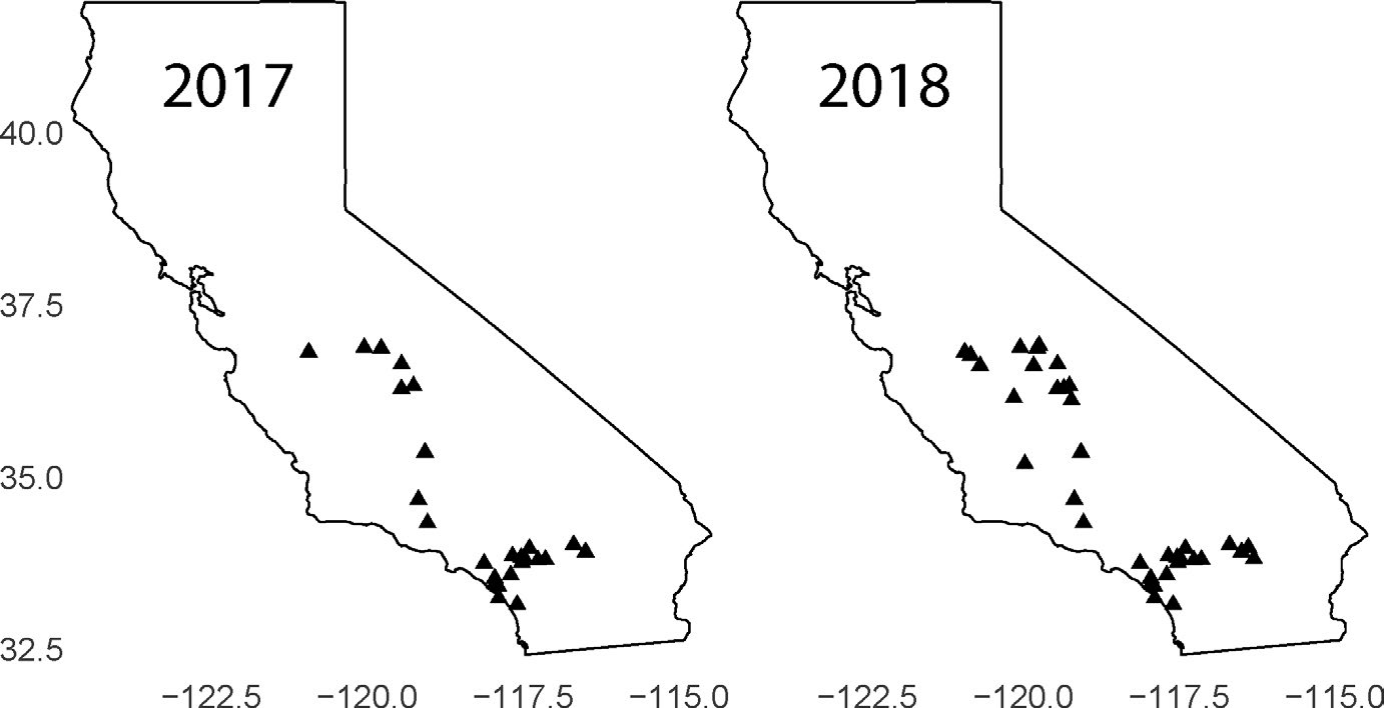
Maps showing the *Datura wrightii* populations visited in 2017 (left; *N* = 25) and 2018 (right; *N* = 35). All populations were initially visited by Hare and Elle (2001) and were located using their published directions. Axes represent latitude and longitude

California *Datura wrightii* populations (*N* = 25; Figure 2a) were visited between 13 July and 5 August 2017. For a random sample of 36 plants within each population (*N*_total_ = 767; nine populations with less than 36 plants, range from 9 to 35 individuals), we noted the arthropods present on each plant, leaf area damaged by herbivores, and the phenotype and number of neighbors (conspecifics within 1m of focal individual). The neighbor cutoff of 1m was selected as this distance should be sufficient for arthropods to recognize plant clusters as separate plants (i.e., plants less than 1m apart will not be recognized as individual plants; Joo et al., 2017). Only one person (JKG) measured herbivory via direct observation to avoid interobserver bias/variation. For populations with fewer than 36 plants, all plants present were sampled. The population‐wide averages of these measurements were used in a prior study (Goldberg et al., 2020), but analyses of variation between individual plants in this dataset have not been previously presented.

In 2018, we visited *D. wrightii* populations (*N* = 35; Figure 2b) twice: once in the spring (30 April–08 May; *N*_plants_ = 772) and again in the summer (31 July–15 August; *N*_plants_ = 927). Two populations (Sequoia National Forest and Tollhouse Grade) had no plants during the spring visitation and one was absent during the summer visitation due to road work (Trimmer Springs Rd.). In the spring, we quantified the leaf area damaged by four major herbivores (*T. notatus*, *L. daturaphila, M. sexta*, and *Epitrix* sp.), and in the summer, we quantified herbivore damage and noted the presence/absence of various arthropod species (herbivores and predators). *Trichobaris compacta* was only present in large enough numbers to be studied in summer 2018.

### 2.3 Statistical analysis

Our raw herbivory data contained many zeroes; therefore, we used a hurdle model approach to address the factors influencing the likelihood of herbivory being present and the intensity of herbivory on focal plants. Hurdle models are two‐part generalized linear models in which the first part (the hurdle) analyzes the data using a logistic regression (positive values are collapsed to 1) and the second part uses a truncated (zeroes excluded) negative binomial regression to look at variation in the positive values only (Rose et al., 2006). For herbivore presence data (which was collected as binary), we used a binomial generalized linear mixed effect model approach. All statistics were conducted in R (R Core Team). Hurdle models were conducted using the glmmTMB() function in the glmmTMB package so that population could be included as a random variable. Population was included as a random factor in each model to account for spatial covariation. Each dependent variable (herbivore/herbivory measures) tested was used in two models: (a) one in which the number of neighbors was included as an explanatory variable; and (b) one in which the frequency of sticky neighbors was included as an explanatory variable (for this analysis, singletons—plants with no neighbors—were excluded). Focal plant phenotype (velvety vs. sticky) was included in each model along with the interaction between it and the continuous explanatory variables. Sticky was always used as the baseline phenotype in our statistical models. In some hurdle models, damage occurrence was too low for variation in the positive values to be analyzed and some GLMMs were unable to converge with the interaction term; thus, this coefficient was excluded for analysis to proceed (noted in Tables 1, 2, 3, 4).

**TABLE 1:** The results of hurdle models comparing the likelihood and magnitude of herbivory with the total number of neighbors plants had. Statistically significant coefficients (*p* < 0.05) are in bold, and nearly significant ones (0.05 < *p* < 0.10) are shown in italics

Observation Period	*N*_obs_	*N*_pop_	*df*	Binary GLM (zeroes versus. positives; “hurdle”)							Truncated Negative Binomial GLM (zeroes excluded)						
				Variance explained by population ± *SD*	β focal plant phenotype		β number of neighbors		β interaction term		Variance explained by population ± *SD*	β focal plant phenotype		β number of neighbors		β interaction term	
					Estimate ± *SE*	*p*‐value	Estimate ± *SE*	*p*‐value	Estimate ± *SE*	*p*‐value		Estimate ± *SE*	*p*‐value	Estimate ± *SE*	*p*‐value	Estimate ± *SE*	*p*‐value
Summer 2017	767	25	756	2.056 ± 1.434	−0.015 ± 0.432	0.972	0.0506 ± 0.213	0.812	0.0796 ± 0.252	0.752	0.157 ± 0.396	**−0.640 ± 0.074**	**<2E−16**	0.0465 ± 0.0316	0.141	−0.0493 ± 0.0443	0.265
Spring 2018	808	33	797	1.432 ± 1.197	0.215 ± 0.265	0.418	0.0380 ± 0.0863	0.66	−0.0294 ± 0.0976	0.763	0.118 ± 0.344	**−0.259 ± 0.0795**	**0.00105**	**0.0751 ± 0.0241**	**0.00187**	**−0.130 ± 0.0316**	**4.39E−05**
Summer 2018	927	35	916	0.891 ± 0.944	0.314 ± 0.285	0.270	−0.0488 ± 0.117	0.677	0.0828 ± 0.137	0.547	0.149 ± 0.385	**−0.611 ± 0.0786**	**7.83E−15**	0.0205 ± 0.0274	0.455	−0.00782 ± 0.0361	0.828
Summer 2017	767	25	756	5.014 ± 2.239	**5.364 ± 0.547**	**<2E−16**	−0.168 ± 0.241	0.486	−0.0243 ± 0.278	0.93	0.174 ± 0.417	**−0.621 ± 0.175**	**0.000403**	0.0487 ± 0.0340	0.153	*−0.198 ± 0.109*	*0.0709*
Spring 2018	808	33	797	1.861 ± 1.364	**3.242 ± 0.410**	**2.53E−15**	−0.0747 ± 0.0916	0.415	*0.333 ± 0.171*	*0.0516*	0.123 ± 0.350	−0.411 ± 0.270	0.127	**0.0736 ± 0.0317**	**0.0204**	−0.0868 ± 0.170	0.61
Summer 2018	927	35	916	1.172 ± 1.083	**3.323 ± 0.318**	**<2E−16**	−0.0399 ± 0.101	0.691	−0.0552 ± 0.140	0.692	0.121 ± 0.347	**−0.911 ± 0.301**	**0.00252**	0.0513 ± 0.0373	0.169	−0.0606 ± 0.151	0.687
Summer 2017	767	25	756	2.100 ± 1.449	**−1.439 ± 0.281**	**2.94E−07**	0.000938 ± 0.135	0.994	0.0394 ± 0.168	0.815	0.176 ± 0.420	**0.491 ± 0.132**	**0.000193**	0.0363 ± 0.0736	0.622	0.00922 ± 0.0813	0.91
Spring 2018	808	33	797	1.57 ± 1.253	−0.411 ± 0.259	0.112	**0.261 ± 0.0977**	**0.00759**	**−0.330 ± 0.109**	**0.00253**	0.171 ± 0.4136	0.134 ± 0.089	0.133	−0.0292 ± 0.0451	0.517	−0.0165 ± 0.0487	0.734
Summer 2018	927	35	916	0.900 ± 0.949	**−0.660 ± 0.241**	**0.00615**	0.0605 ± 0.101	0.551	0.0418 ± 0.119	0.725	0.191 ± 0.437	−0.0156 ± 0.126	0.901	−0.0987 ± 0.0644	0.126	**0.153 ± 0.073**	**0.0358**
Summer 2017	767	25	756	2.723 ± 1.650	**−3.338 ± 0.833**	**6.12E−05**	−0.300 ± 0.395	0.447	0.423 ± 0.411	0.303	0.0215 ± 0.147	0.343 ± 0.476	0.472	0.0441 ± 0.195	0.821	−0.0807 ± 0.205	0.694
Spring 2018	808	33	797	6.518 ± 2.553	*−2.604 ± 1.370*	*0.0573*	0.426 ± 0.842	0.613	−0.259 ± 0.851	0.7612	Model did not converge (low occurrence)						
Summer 2018	927	35	916	2.864 ± 1.692	**−2.552 ± 0.486**	**1.51E−07**	−0.230 ± 0.188	0.219	0.0618 ± 0.203	0.761	0.0777 ± 0.279	0.205 ± 0.270	0.447	−0.0632 ± 0.118	0.592	0.104 ± 0.121	0.392
Summer 2017	767	25	756	1.698 ± 1.303	0.306 ± 0.319	0.337	−0.0456 ± 0.164	0.781	0.104 ± 0.197	0.597	0.0657 ± 0.256	**−0.332 ± 0.148**	**0.0247**	**−0.408 ± 0.130**	**0.00175**	**0.440 ± 0.139**	**0.00151**
Spring 2018	808	33	797	125.6 ± 11.21	−0.169 ± 1.510	0.911	0.0145 ± 0.413	0.972	1.970 ± 1.231	0.109	Model did not converge (low occurrence)						
Summer 2018	927	35	916	0.554 ± 0.744	0.0332 ± 0.307	0.914	**0.387 ± 0.168**	**0.0213**	−0.0816 ± 0.206	0.693	6.003E−09 ± 7.748E−05	−0.169 ± 0.181	0.35	−0.128 ± 0.130	0.326	0.106 ± 0.158	0.503
Summer 2018	927	35	916	4.361 ± 2.088	**−1.193 ± 0.355**	**0.000776**	−0.0296 ± 0.149	0.843	0.0273 ± 0.170	0.873	0.109 ± 0.329	**0.323 ± 0.132**	**0.014**	0.0487 ± 0.0517	0.346	−0.0567 ± 0.0627	0.366

**TABLE 2:** The results of hurdle models comparing the likelihood and magnitude of herbivory with the frequency of sticky plants in the immediate neighborhood. Statistically significant coefficients (*p* < 0.05) are in bold, and nearly significant ones (0.05 < *p* < 0.10) are shown in italics

Type of damage observed	Observation Period	*N*_obs_	*N*_pop_	*df*	Binary GLM (zeroes vs. positives; "hurdle")							Truncated Negative Binomial GLM (zeroes excluded)						
					Variance explained by population ± *SD*	β focal phenotype		β phenotype frequency		β interaction term		Variance explained by population ± *SD*	β focal phenotype		β neighborhood		β interaction term	
						Estimate ± *SE*	*p*‐value	Estimate ± *SE*	*p*‐value	Estimate ± *SE*	*p*‐value		Estimate ± *SE*	*p*‐value	Estimate ± *SE*	*p*‐value	Estimate ± *SE*	*p*‐value
Any	Summer 2017	378	23	367	2.533 ± 1.591	**1.391 ± 0.677**	**0.0397**	0.690 ± 0.780	0.377	**−2.672 ± 1.113**	**0.0164**	0.126 ± 0.355	**−0.672 ± 0.106**	**2.11E−10**	0.142 ± 0.102	0.165	−0.265 ± 0.165	0.109
	Spring 2018	464	32	453	1.536 ± 1.239	0.223 ± 0.392	0.569	0.242 ± 0.516	0.639	−0.111 ± 0.650	0.864	0.0618 ± 0.249	**−0.727 ± 0.106**	**7.84E−12**	−0.140 ± 0.130	0.281	0.252 ± 0.201	0.21
	Summer 2018	516	30	505	1.18 ± 1.086	0.425 ± 0.501	0.396	−0.262 ± 0.589	0.656	0.749 ± 0.705	0.287	0.143 ± 0.377	**−0.509 ± 0.118**	**1.39E−05**	*0.236 ± 0.131*	*0.0715*	−0.0925 ± 0.178	0.603
mirid suckfly (*Tupiochoris notatus*)	Summer 2017	378	23	367	5.587 ± 2.364	**6.241 ± 1.015**	**7.94E−10**	1.032 ± 0.858	0.229	*−1.944 ± 1.020*	*0.0567*	0.150 ± 0.388	**−1.075 ± 0.194**	**3.24E−08**	*0.192 ± 0.107*	*0.0733*	0.232 ± 0.285	0.416
	Spring 2018	464	32	453	2.004 ± 1.416	**4.986 ± 0.715**	**3.13E−12**	0.868 ± 0.537	0.106	**−3.285 ± 1.024**	**0.00134**	0.266 ± 0.516	**−0.704 ± 0.357**	**0.0483**	−0.280 ± 0.173	0.106	0.0127 ± 0.551	0.982
	Summer 2018	516	30	505	0.976 ± 0.988	**3.145 ± 0.460**	**8.25E−12**	*−0.786 ± 0.475*	*0.0981*	−0.0532 ± 0.690	0.939	0.107 ± 0.327	**−0.860 ± 0.321**	**0.00747**	0.0140 ± 0.168	0.934	−0.294 ± 0.572	0.607
leaf beetles (*Lema daturaphila*)	Summer 2017	378	23	367	3.284 ± 1.812	**−1.081 ± 0.437**	**0.0133**	0.523 ± 0.487	0.283	−0.326 ± 0.653	0.618	0.0973 ± 0.312	**0.478 ± 0.195**	**0.0143**	−0.214 ± 0.237	0.367	−0.132 ± 0.285	0.642
	Spring 2018	464	32	453	1.662 ± 1.289	**−0.836 ± 0.367**	**0.0229**	0.7696 ± 0.5224	0.141	−0.355 ± 0.651	0.586	0.0650 ± 0.255	0.0702 ± 0.141	0.617	0.289 ± 0.183	0.114	−0.305 ± 0.234	0.193
	Summer 2018	516	30	505	1.037 ± 1.018	−0.406 ± 0.395	0.304	0.267 ± 0.472	0.571	−0.308 ± 0.580	0.596	0.290 ± 0.539	0.313 ± 0.194	0.106	0.289 ± 0.252	0.252	0.0175 ± 0.275	0.949
flea beetles (*Epitrix* sp.)	Summer 2017	378	23	367	4.22 ± 2.054	*−2.326 ± 1.316*	*0.0772*	−0.589 ± 1.702	0.729	−1.179 ± 1.754	0.502	3.213E−10 ± 1.792E−05	−1.425 ± 1.875	0.447	−2.710 ± 3.163	0.392	2.337 ± 3.169	0.461
	Spring 2018	464	32	453	Model did not converge (very low occurrence)													
	Summer 2018	516	30	505	3.956 ± 1.989	**−2.398 ± 0.701**	**0.000618**	0.512 ± 1.038	0.622	0.262 ± 1.130	0.816	0.120 ± 0.347	*0.842 ± 0.454*	*0.0639*	0.688 ± 0.724	0.342	−0.888 ± 0.770	0.249
tobacco hornworm (*Manduca sexta*)	Summer 2017	378	23	367	1.168 ± 1.081	−0.238 ± 0.535	0.657	*−1.213 ± 0.621*	*0.0507*	*1.371 ± 0.826*	*0.0971*	0.0563 ± 0.237	**0.735 ± 0.367**	**0.0452**	0.261 ± 0.436	0.549	−0.521 ± 0.548	0.342
	Spring 2018	464	32	453	Model did not converge (very low occurrence)													
	Summer 2018	516	30	505	0.979 ± 0.989	−1.331 ± 0.934	0.154	*−1.679 ± 1.003*	*0.094*	1.814 ± 1.155	0.116	1.93E−09 ± 4.393E−05	−0.557 ± 0.369	0.131	−0.631 ± 0.396	0.111	0.376 ± 0.521	0.471
stem‐boring weevils (*Trichobaris* sp.)	Summer 2018	516	30	505	5.117 ± 2.262	*−0.966 ± 0.559*	*0.0839*	0.521 ± 0.714	0.464	0.0836 ± 0.837	0.92	0.0663 ± 0.258	−0.128 ± 0.204	0.529	−0.146 ± 0.212	0.492	**0.673 ± 0.274**	**0.014**

**TABLE 3:** The results of analyses comparing the likelihood of observing various arthropods on a focal plant and the total number of neighbors that plant had. Statistically significant coefficients (*p* < 0.05) are in bold, and nearly significant ones (0.05 < *p* < 0.10) are shown in italics

Type of observation	Observation period	*N*_obs_	*N*_pop_	*df*	Variance explained by population ± *SD*	β focal phenotype		β number of neighbors		β interaction term	
						Estimate ± *SE*	*p*‐value	Estimate ± *SE*	*p*‐value	Estimate ± *SE*	*p*‐value
mirid suckfly (*Tupiochoris notatus*) nymphs and/or adults	Summer 2017	767	25	756	2.537 ± 1.593	**−4.425 ± 0.448**	**<2E−16**	−0.181 ± 0.158	0.252	0.227 ± 0.244	0.353
	Summer 2018	927	35	916	1.156 ± 1.075	**−4.086 ± 0.496**	**<2E−16**	0.041 ± 0.096	0.673	−0.114 ± 0.236	0.629
leaf beetles (*Lema daturaphila*) adults and/or larvae	Summer 2017	767	25	756	2.656 ± 1.63	**1.212 ± 0.351**	**0.000559**	−0.134 ± 0.192	0.484	0.022 ± 0.226	0.922
	Summer 2018	927	35	916	1.575 ± 1.255	0.413 ± 0.343	0.229	−0.124 ± 0.161	0.44	0.084 ± 0.184	0.65
flea beetles (*Epitrix* sp.)	Summer 2017	767	25	756	1.347 ± 1.160	*1.640 ± 0.870*	*0.0593*	−0.013 ± 0.518	0.98	−0.430 ± 0.570	0.452
	Summer 2018	927	35	916	8.078 ± 2.842	1.143 ± 0.812	0.159	0.029 ± 0.279	0.917	−0.139 ± 0.306	0.65
tobacco hornworm (*Manduca sexta*) larvae of any instar	Summer 2017	767	25	756	1.473 ± 1.213	−0.100 ± 0.651	0.877	0.111 ± 0.315	0.724	−0.197 ± 0.391	0.615
	Summer 2018	927	35	916	4.085 ± 2.021	0.653 ± 0.900	0.468	−0.300 ± 0.629	0.633	0.049 ± 0.684	0.943
stem‐boring weevils (*Trichobaris* sp.)	Summer 2018	927	35	916	2.089 ± 1.445	−0.326 ± 0.652	0.617	−0.396 ± 0.414	0.339	0.062 ± 0.496	0.9
*Manduca sexta* eggs	Summer 2017	767	25	756	2.334 ± 1.528	*−1.633 ± 0.903*	*0.0706*	*−1.087 ± 0.601*	*0.0705*	EXCLUDED FROM MODEL	
	Summer 2018	927	35	916	1.001 ± 1.001	−0.948 ± 0.707	0.18	−0.293 ± 0.343	0.394	0.122 ± 0.460	0.79
*Lema daturaphila* eggs	Summer 2017	767	25	756	1.411 ± 1.188	**0.702 ± 0.299**	**0.0188**	−0.240 ± 0.172	0.163	0.136 ± 0.203	0.5
	Summer 2018	927	35	916	1.342 ± 1.158	0.063 ± 0.342	0.854	*−0.381 ± 0.201*	*0.0568*	0.129 ± 0.231	0.576
arthropod predators (hemipterans, spiders)	Summer 2017	767	25	756	MODEL FAILED TO CONVERGE						
	Summer 2018	927	35	916	0.476 ± 0.670	−0.183 ± 0.398	0.645	0.183 ± 0.127	0.149	**−0.481 ± 0.220**	**0.0287**

**TABLE 4:** The results of analyses comparing the likelihood of observing various arthropods on a focal plant and the frequency of sticky plants in the immediate neighborhood. Statistically significant coefficients (*p* < 0.008) are in bold, nearly significant ones (0.008 < *p* < 0.10) are shown, and all nonsignificant coefficients (*p* > 0.10) are shown as NS

Type of observation	Observation period	*N*_obs_	*N*_pop_	*df*	Variance explained by population ± *SD*	β focal plant phenotype		β sticky neighbor frequency		β interaction term	
						Estimate ± *SE*	*p*‐value	Estimate ± *SE*	*p*‐value	Estimate ± *SE*	*p*‐value
mirid suckfly (*Tupiochoris notatus*) nymphs and/or adults	Summer 2017	378	23	367	3.019 ± 1.738	**−5.723 ± 0.796**	**6.12E−13**	**−1.531 ± 0.658**	**0.02**	**2.978 ± 0.963**	**0.00198**
	Summer 2018	516	30	453	1.234 ± 1.111	**−5.297 ± 0.874**	**1.37E−09**	0.341 ± 0.486	0.483	*1.945 ± 1.110*	*0.0797*
leaf beetles (*Lema daturaphila*) adults and/or larvae	Summer 2017	378	23	367	2.815 ± 1.678	0.182 ± 0.579	0.754	−1.120 ± 0.687	0.103	**1.942 ± 0.837**	**0.0203**
	Summer 2018	516	30	453	1.888 ± 1.374	−0.190 ± 0.555	0.733	*−1.363 ± 0.756*	*0.0713*	*1.580 ± 0.899*	*0.0789*
flea beetles (*Epitrix* sp.)	Summer 2017	378	23	367	1.917 ± 1.384	−0.290 ± 1.062	0.785	−1.557 ± 1.868	0.404	2.610 ± 2.060	0.205
	Summer 2018	516	30	453	12.73 ± 3.568	0.450 ± 1.026	0.661	−3.043 ± 3.631	0.402	1.186 ± 3.995	0.767
tobacco hornworm (*Manduca sexta*) larvae of any instar	Summer 2017	378	23	367	6.729E−14 ± 2.594E−07	−0.579 ± 0.634	0.361	−0.673 ± 0.720	0.35	EXCLUDED FROM MODEL	
	Summer 2018	516	30	453	15.73 ± 3.967	−0.773 ± 1.317	0.557	−314.0 ± 319.9	0.326	314.57 ± 319.85	0.325
stem‐boring weevils (*Trichobaris* sp.)	Summer 2018	516	30	453	6.066 ± 2.463	1.079 ± 2.450	0.66	0.138 ± 2.740	0.96	−1.800 ± 3.657	0.623
*Manduca sexta* eggs	Summer 2017	378	23	367	2.779 ± 1.667	−164.4 ± 1,448	0.91	0.743 ± 2.049	0.717	EXCLUDED FROM MODEL	
	Summer 2018	516	30	453	3.409 ± 1.846	−0.666 ± 1.045	0.525	0.374 ± 1.160	0.747	EXCLUDED FROM MODEL	
*Lema daturaphila* eggs	Summer 2017	378	23	367	2.129 ± 1.459	−0.170 ± 0.488	0.727	**−1.413 ± 0.611**	**0.0207**	**2.360 ± 0.750**	**0.00165**
	Summer 2018	516	30	453	1.761 ± 1.327	−0.777 ± 0.596	0.192	−1.127 ± 0.764	0.14	**2.199 ± 0.915**	**0.0163**
arthropod predators (Hemipterans, spiders)	Summer 2017	378	23	367	0.5058 ± 0.7114	**−1.669 ± 0.560**	**0.00287**	0.0307 ± 0.490	0.95	*1.389 ± 0.776*	*0.0737*
	Summer 2018	516	30	453	0.9366 ± 0.9678	**−1.874 ± 0.775**	**0.0156**	0.164 ± 0.695	0.814	1.255 ± 1.054	0.2338

## 3 RESULTS

**FIGURE 3:**
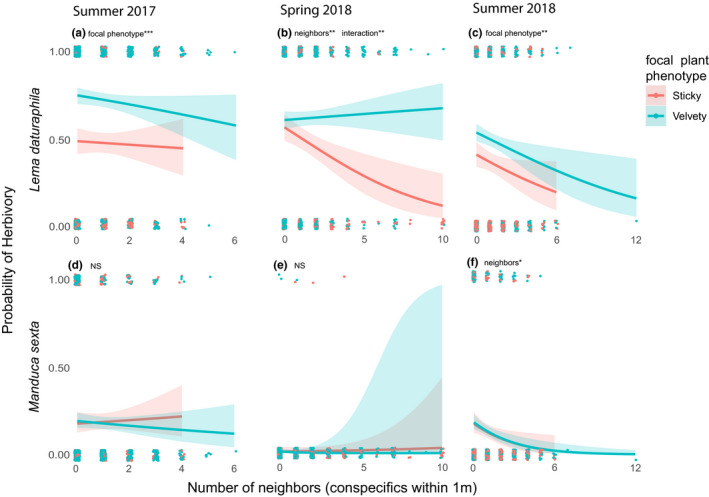
Results of the “hurdle” portion of hurdle models that test the ability of focal plant phenotype, total number of neighboring conspecifics, and the interaction of them to predict the likelihood of herbivory being present on focal plants. The top row (panels a, b, and c) represents the results of models with the likelihood of *Lema daturaphila* damage as the response variable, while the bottom row (panels d, e, and f) shows the likelihood of *Manduca sexta* damage as the response variable. Significant coefficients in each model are noted along with * denoting the *p*‐value (* 0.01 < *p* < 0.05; ** 0.001 < *p* < 0.01, *** *p* < 0.001). Exact *p*‐values and tests statistics are located in Table 1

**FIGURE 4:**
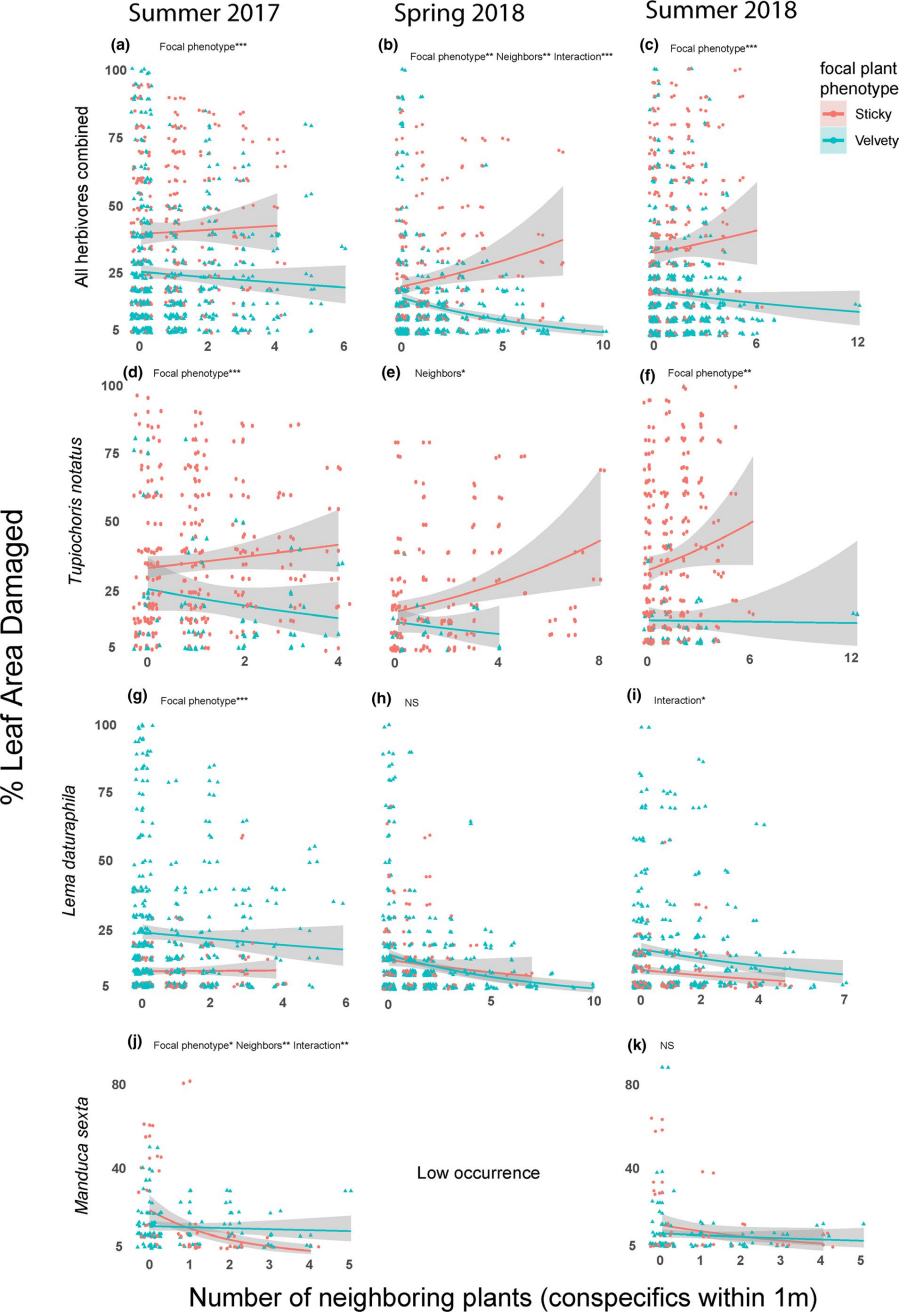
Results of the truncated negative binomial glm portion of hurdle models. These panels show the results of models predicting the magnitude of herbivory on focal plants where damage was present. Each row of figures shows a different response variable: Panels a/b/c show all herbivore damage combined, panels D/E/F show *Tupiochoris notatus* damage, panels g/h/i show *Lema daturaphila* damage, and the bottom row (panels j/k) shows *Manduca sexta* damage. Significant coefficients in each model are noted along with * denoting the *p*‐value (* 0.01 < *p* < 0.05; ** 0.001 < *p* < 0.01, *** *p* < 0.001). Exact *p*‐values and tests statistics are located in Table 1

**FIGURE 5:**
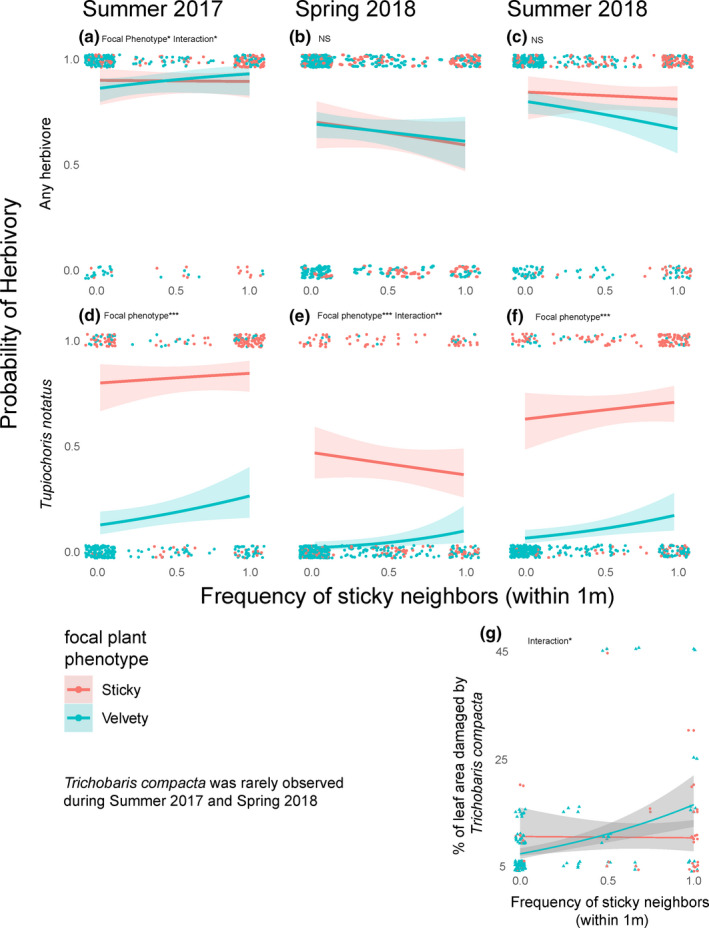
Results of analysis comparing the likelihood of a plant being damaged by any herbivore (top row, panels a/b/c) or *Tupiochorus notatus* (second row, panels d/e/f) and the frequency of sticky *Datura wrightii* plants within 1 m of focal plants. We also show the relationship between the frequency of sticky neighbors and the leaf area damaged by Trichobaris compacta (panel g). Significant coefficients in each model are noted along with * denoting the *p*‐value (* 0.01 < *p* < 0.05; ** 0.001 < *p* < 0.01, *** *p* < 0.001). Exact *p*‐values and tests statistics are in Table 2

**FIGURE 6:**
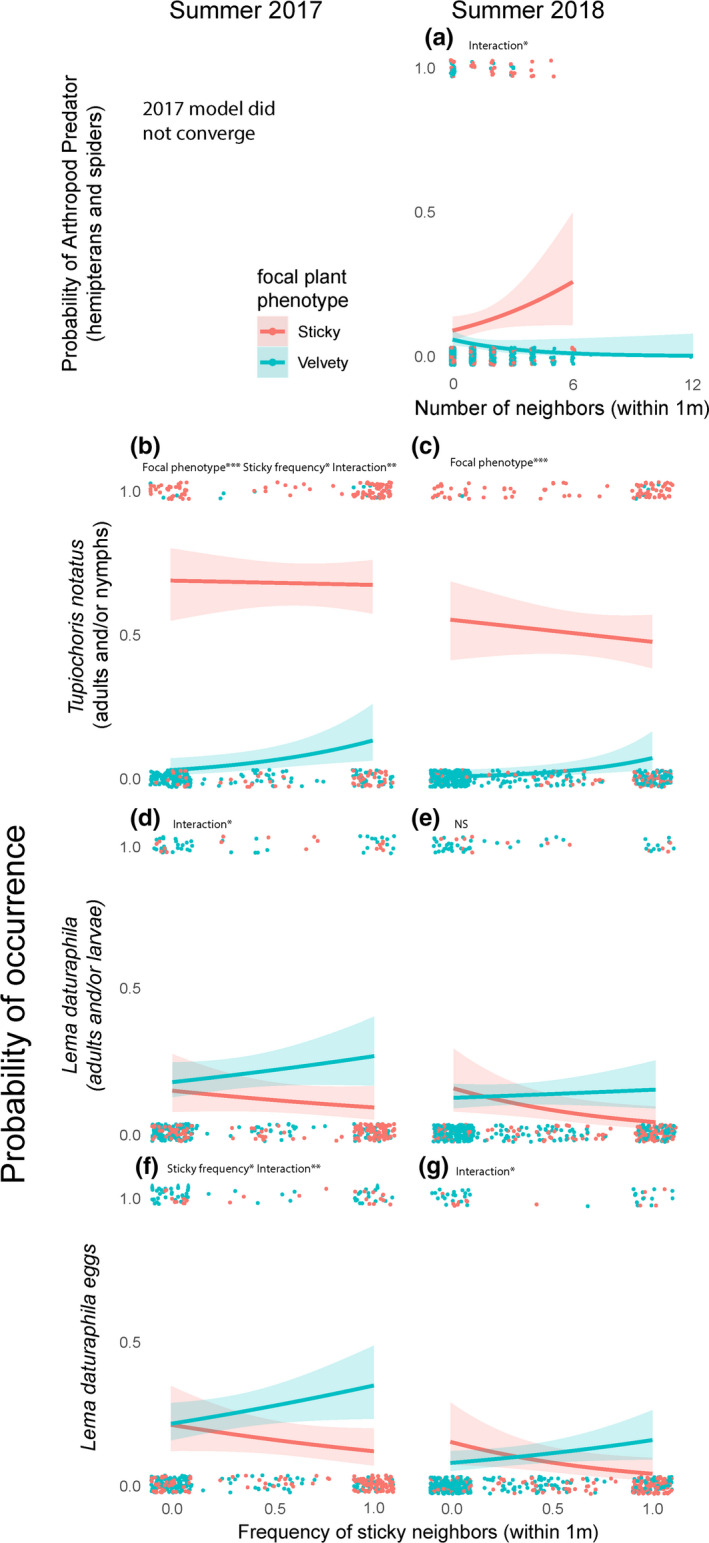
Results of analysis using both neighborhood metrics (total number of numbers or frequency of sticky neighbors) to predict the presence/absence of various arthropods on dimorphic *Datura wrightii* plants. The top panel (a) shows the relationship between total number of neighbors and the likelihood of a predatory arthropod being present on focal plants (data shown in Table 3). The remaining panels show the relationship between frequency of sticky neighbors and the likelihood of *Tupiochoris notatus* individuals (panels b/c), *Lema daturaphila* individuals (panels d/e), or *Lema daturaphila* eggs (panels f/g) being present on focal plants. Test statistics and *p*‐values for panels b–g are shown in Table 4. Significant coefficients in each model are noted along with * denoting the *p*‐value (* 0.01 < *p* < 0.05; ** 0.001 < *p* < 0.01, *** *p* < 0.001)

Results of all models are summarized in Tables 1, 2, 3, 4. Table 1 shows the results of hurdle models examining the relationship between herbivore damage and the total number of neighboring plants (selected results are visualized in Figures 3 and 4). Table 2 shows the results of hurdle models looking at the relationship between herbivory and the frequency of sticky plants in the immediate neighborhood (selected results shown in Figure 5). Table 3 shows the results from GLMMs looking at the relationship between arthropod occurrence and total number of neighboring plants, and Table 4 shows results of models examining the relationship between arthropod occurrence and the sticky neighbor frequency (selected results from both tables are shown in Figure 6).

Overall, focal plant phenotype was better at predicting the presence or absence of herbivore damage than was variation in neighborhood density or composition (15 out of 32 β_focal plant phenotype_ coefficients were significant vs. only 2 β_neighbors/sticky frequency_ and 3 β_interaction_; Tables 1 and 2). This was also the case for truncated GLMs predicting the magnitude of herbivory (16 out of 32 β_focal plant phenotype_ coefficients were significant vs. only 3 β_neighbors/sticky frequency_ and 4 β_interaction_; Tables 1 and 2). Focal phenotype was able to predict the likelihood or magnitude of herbivory in at least one timepoint for all herbivores included in our study (Tables 1 and 2).

We considered significant (*p* <0.05) β_neighbors/sticky frequency_ or β_interaction_ evidence of associational effects occurring at that timepoint. Consistent associational effects (occurring at all timepoints) were not observed for any herbivore, but they were observed in at least one timepoint for each herbivore except flea beetles (*Epitrix* sp.). In both summer 2017 and 2018, only the focal phenotype was able to predict the likelihood of *L. daturaphila* damage with velvety plants being more likely to be damaged (summer 2017: β_focal plant phenotype_ = −1.439, *p* < 0.0001, Figure 3a, Table 1; summer 2018: β_focal plant phenotype_ = −0.660, *p* = 0.00615, Figure 3c, Table 1). In spring 2018, we observed that sticky *D. wrightii* plants were less likely to be damaged by *L. daturaphila* when they had a larger number of neighbors (β_neighbors_ = 0.261_,_ *p* = 0.00759; Figure 3b, Table 1), but that this was not the case for velvety plants (β_interaction_ = −0.330_,_ *p* = 0.00253; Figure 3b, Table 1). In summer 2018, we observed that plants with more neighbors were less likely to be damaged by *M. sexta* (β_neighbors_ = 0.387_,_ *p* = 0.0213; Figure 3f, Table 1) and that this relationship was the same for both *D. wrightii* phenotypes (β_focal plant phenotype_ = 0.0332, *p* = 0.914; β_interaction_ = −0.0816_,_ *p* = 0.693; Figure 3f, Table 1).

The number of neighboring plants was found to influence the magnitude of herbivory by multiple insect species at different timepoints (Table 1; Figure 4). When all forms of herbivory were combined, we found that in the spring of 2018, sticky plants received more damage than velvety plants (β_focal plant phenotype_ = −0.259, *p* = 0.00105**; Figure 4b) and that the damage they received increased with the number of neighbors (β_neighbors_ = 0.0751_,_ *p* = 0.00187**; Figure 4b). Furthermore, velvety plants received less damage when they had more neighbors (β_interaction_ = −0.130_,_ *p* < 0.0001; Figure 4b). When we considered each herbivore separately, we found that the number of neighboring plants had a significant effect for three species of herbivore at three different times: *Tupiochoris notatus* (spring 2018; Figure 4e), *Lema daturaphila* (summer 2018; Figure 4i), and *Manduca sexta* (summer 2017; Figure 4j). *T. notatus* was positively correlated with the number of neighbors in spring 2018, indicating that plants in more numerous clusters received more damage than singletons without any (β_neighbors_ = 0.0736_,_ *p* = 0.02*; Figure 4e). The absence of a significant interaction term (β_interaction_ = −0.130. *p* = 0.610) suggests that this relationship applies to both trichome phenotypes, although this does not visually appear to be the case (Figure 4e). In both summer 2017 and 2018, *T. notatus* damaged more leaf area on sticky plants than velvety ones (summer 2017: β_focal plant phenotype_ = −0.621, *p* = 0.00040***, Figure 4d; summer 2018: β_focal plant phenotype_ = −0.911, *p* = 0.00252**, Figure 4f), but damage was not associated with the number of neighboring plants (Table 1). *Lema daturaphila* damaged more leaf area on velvety plants than sticky ones in summer 2017 (β_focal plant phenotype_ = 0.491, *p* = 0.00019***; Figure 4g), but this was not the case during any measurement in 2018 (spring: β_focal plant phenotype_ = −0.621, *p* = 0.133, Figure 4h; summer: β_focal plant phenotype_ = −0.0156, *p* = 0.901, Figure 4i). In summer 2018, we found a significant interaction term (β_interaction_ = 0.153_,_ *p* = 0.0358*; Figure 4i) suggesting that there is a negative relationship with the number of neighbors for velvety plants, but not for sticky ones (β_neighbors_ = −0.0987, *p* = 0.126). In summer 2017, we found that all three coefficients were significant for *Manduca sexta* (β_focal plant phenotype_ = −0.332, *p* = 0.0247*; β_neighbors_ = −0.408 *p* = 0.00175**; β_interaction_ = 0.440, *p* = 0.00151**, Figure 4j). This suggests that sticky singletons (plants with no neighbors) had more leaf area damaged than velvety singletons, that sticky plants with more neighbors received less damage from *M. sexta*, and that the number of neighbors was not related to the damage received by velvety plants. In summer 2018, we found that neither focal plant phenotype nor the number of neighboring plants was able to predict the leaf area damaged by *M. sexta* (β_focal plant phenotype_ = −0.169, *p* = 0.350; β_neighbors_ = −0.128, *p* = 0.326; β_interaction_ = 0.106, *p* = 0.503, Figure 4k).

When we excluded singletons to examine the effect of neighborhood composition (i.e., the frequency of the sticky phenotype within 1m of the focal plant; Table 2), we found only two cases where it was able to predict the likelihood of herbivore on focal plants: for the presence of any damage in summer 2017 (Figure 5a) and the likelihood of *T. notatus* damage in spring 2018 (Figure 5e). In summer 2017, we found that sticky plants with all‐velvety neighbors were more likely to be damaged by any herbivore than velvety plants with all‐velvety neighbors (β_focal plant phenotype_ = 1.391, *p* = 0.0397*; Figure 5a) and that the likelihood of any herbivory being present on velvety plants increased with the sticky frequency in the local neighborhood (β_interaction_ = −2.672, *p* = 0.0164*; Figure 5a), but that the frequency of sticky neighbors did influence the likelihood of herbivory on sticky focal plants (β_neighbors_ = 0.069, *p* = 0.377; Figure 5a). In spring 2018, we found that the likelihood of *T. notatus* herbivory being present on velvety plants increased with the frequency of neighboring sticky plants (β_interaction_ = −3.328, *p* = 0.00134*; Figure 5e), but that this relationship was not significant for sticky plants (β_neighbors_ = 0.868, *p* = 0.106; Figure 5e). Sticky focal plants were always more likely than velvety ones to be damaged by *T. notatus* (summer 2017: β_neighbors_ = 6.241_,_ *p* < 0.001***; Figure 5d; spring 2018: β_neighbors_ = 4.986, *p* < 0.001***; Figure 5e; β_neighbors_ = 3.145, *p* < 0.001***; Figure 5f). The frequency of sticky neighbors was only able to predict the magnitude of herbivory by one herbivore: *Trichobaris compacta* (Table 2; Figure 5g). This herbivore was only observed in the summer of 2018, and the leaf area damaged by this herbivore increased with the frequency of sticky plants neighboring velvety focal plants (β_interaction_ = 0.673, *p* = 0.0140*; Figure 5g), but not for sticky focal plants (β_neighbors_ = −0.146, *p* = 0.492; Figure 5g).

When we looked at the ability of the number of neighboring plants to predict the likelihood of arthropod presence, we found only one significant relationship: for arthropod predators (hemipterans and spiders) in summer 2018 (Table 3; Figure 6a). For this model, we found a significant interaction term (β_interaction_ = −0.481, *p* = 0.0287*; Figure 6a) indicating that the relationship between the number of neighbors and the likelihood of an arthropod predator being present on a plant differs for sticky and velvety focal plants. The likelihood of predators being present on a sticky focal plant was not related to the number of neighboring plants (β_neighbors_ = 0.183, *p* = 0.149; Figure 6a); thus, we interpret the significant interaction term as indicative of a negative relationship between the likelihood of predator presence and the number of neighboring plants for velvety focal plants (Table 3; Figure 6a).

We found multiple cases of the frequency of sticky neighboring plants predicting the presence/absence of arthropods (Table 4; Figure 6b–g): *T. notatus* individuals (adults/nymphs; Figure 6b,c), *L. daturaphila* individuals (adults/all larval instars; Figure 6d,e), and *L. daturaphila* eggs (Figure 6f,g). In the summer of 2017, all variables were able to predict the likelihood of *T. notatus* being present on a given focal plant (β_focal plant phenotype_ = −5.723, *p* < 0.001***; β_neighbors_ = −1.531, *p* = 0.020*; β_interaction_ = 2.978, *p* = 0.00198**, Figure 6b). We interpret this as meaning that sticky focal plants are more likely to have *T. notatus* on them than velvety focal plants, that sticky focal plants with a greater frequency of sticky neighbors are less likely to have *T. notatus* on them, and that velvety focal plants are more likely to have *T. notatus* individuals on them when they have a higher frequency of sticky neighboring plants (Figure 6b). In the summer of 2018, only the focal plant phenotype was able to predict the likelihood of *T. notatus* being present, with sticky focal plants having a far greater likelihood than velvety focal plants (β_focal plant phenotype_ = −5.297, *p* < 0.001***; Figure 6c, Table 4). For *L. daturaphila* individuals (both adult beetles and larvae of all instars), we found a significant interaction term in summer 2017 (β_interaction_ = 1.942, *p* = 0.0203*; Figure 6d), indicating that this herbivore is more likely to be present on velvety focal plants with sticky neighbors than velvety focal plants surrounded by other velvety plants. For *L. daturaphila* eggs, we found that both that the frequency of sticky neighbors influenced the likelihood of their presence on both sticky and velvety focal plants (β_neighbors_ = −1.413_,_ *p* = 0.0207*; β_interaction_ = 2.360, *p* = 0.00165**, Figure 6f). This indicates that the likelihood of *L. daturaphila* eggs being present on a sticky focal plant decreased with the frequency of sticky neighbors, while increasing for the velvety focal plants. In summer 2018, we only observed a significant interaction term (β_interaction_ = 2.199, *p* = 0.0163*; Figure 6g) indicating that the frequency of sticky neighbors only predicted the likelihood of eggs being present on velvety focal plants at this time.

## 4 DISCUSSION

Our results are, for the most part, consistent with previous studies of herbivore preferences for the *D. wrightii* morphs (Hare & Elle, 2002). Flea beetles only rarely were found to attack sticky plants, *T. notatus* was consistently more likely to damage the sticky morph (and the sticky morph received more damage than velvety morphs), and *M. sexta* was equally likely to feed on both morphs. We found that *L. daturaphila* was sometimes more likely to feed on velvety plants, which was not previously observed but also not unprecedented given that sticky plants have been shown to inhibit their grown rate (Hare & Elle, 2002). Our data further show that geographic heterogeneity in abundance and damage to plants is often strong for some herbivores (flea beetles, *M. sexta*, and *T. notatus* especially). We also found some signs of the local plant neighborhood as impacting the likelihood and magnitude of herbivory on our focal *D. wrightii* individuals.

Perhaps the most striking pattern of associational effects that we observed is that velvety plants surrounded by sticky plants appear to be more susceptible to multiple forms of herbivory. Our data show a clear “spillover pattern” in which velvety plants in predominantly sticky patches are more likely to be attacked by *T. notatus*, an herbivore that primarily infests the sticky *D. wrightii* morph (Figures 5e and 6b). We also found that velvety *D. wrightii* surrounded by the sticky morph are more susceptible to infestation by *L. daturaphila* (Figure 6d,f,g) and are more heavily damaged by *T. compacta* weevils (Figure 5g). These findings—which clearly show that velvety plants do not receive a locally rare advantage—go against the hypothesis that associational effects would underlie NFDS on the *D. wrightii* trichome dimorphism. Indeed, these results are more consistent with the predictions of apparent competition, in which asymmetric effects of a shared natural enemy drive certain members of an assemblage extinct while allowing others to persist (Holt & Bonsall, 2017). In other words, the apparent preference of *L. daturaphila* for velvety plants is causing positive frequency‐dependent herbivory in favor of the sticky morph. Indeed, should the observed susceptibility to herbivores by rare velvety plants result in a fitness reduction, one would expect this morph to be extirpated from predominantly sticky populations (Bonsall & Hassel, 1997). This extirpation has not been observed. Instead over the past two decades, the two morphs have continued to coexist (Goldberg et al., 2020). Taken together, our studies suggest two possibilities for why NFDS occurs over time among populations (i.e., the velvety morph increases when rare [Goldberg et al., 2020]), despite velvety plants receiving more damage when locally rare. The first is that the scale at which we measured herbivory in this study was too small to capture the full extent of herbivory on the two trichome morphs within populations in our study system. This could be compounded by variation in the density of *D. wrightii* (see below). The second is that the negative effect of herbivory might vary with age, being greater in small, young individuals and less in established individuals such as the focal individuals used in this study. In other words, the increase in herbivory we observed on rare velvety plants may not be enough to drive a significant reduction in fitness. This is supported by previous studies showing that *D. wrightii* can be exceptionally tolerant to herbivory, withstanding large portions of vegetation tissue being damaged without a reduction in seed production (Elle et al., 1999; Hare & Elle, 2002).

The effect of neighboring plants on the susceptibility of the sticky trichome morph was more varied than the effect on velvety plants. Our data show that the total number of neighboring plants was usually a better predictor of herbivory/herbivore presence (Figures 3b,f and 4b,e,j) than the frequency of sticky neighbors (Figure 6b). Sticky plants with more neighbors (of any phenotype) were less likely to be damaged by *L. daturaphila* (Figure 3b) or *M. sexta* (Figure 3f). We interpret this as an effect of herbivores spreading out across large clusters of plants and avoiding lower quality hosts (which in our system is the sticky phenotype, presumably due to noxious compounds in the exudate) in favor of higher quality host plants (velvety, in the *D. wrightii* system). This finding is further reinforced by the observation that *M. sexta* damages less leaf area on sticky plants with more neighbors (Figure 4j). This effect only appears to apply to the herbivores which infest both *D. wrightii* trichome morphs as *T. notatus* (which strongly favors the sticky morph) damaged more leaf area on plants in larger clusters (Figure 4e). Given that the predictive variable in these cases was the total number of neighbors of both trichome phenotypes, it is likely that this is a density‐dependent process rather than a morph frequency‐dependent process; however, because we did not strictly quantify the density of *D. wrightii* plants (neighborhood area in our study varies based upon the size of the focal plant), more carefully controlled observations/experimentation are required to confirm the effect of population density on herbivory in this system.

We also found evidence that likelihood of arthropod predators being found on a plant was dependent on the number of neighboring plants (Figure 6a). In this case, velvety plants were less likely to have predators on them when they had more neighbors. This finding is interesting because it suggests that the efficacy of plant indirect defenses may be density‐dependent to some extent. Arthropod predators may avoid lower quality host plants in large clusters where higher quality options exist. Our own data show that predators are more likely to occur on sticky plants than velvety ones (Table 4, rows 16/17; not shown in any figure) and there is further evidence for this preference in the literature for other tritrophic systems (Vasconellos‐Neto et al., 2007). However, this finding contradicts prior research on the *D. wrightii* system which showed that the sticky morph has less effective indirect defenses than the velvety morph (Gassmann & Hare, 2005). These data highlight the need for more detailed observations of the *D. wrightii*‐associated predator community and how predator behavior may provide asymmetric benefits to the two *Datura wrightii* trichome morphs in nature.

In summary, we found evidence that associational effects between neighboring conspecifics can occur within dimorphic populations of *Datura wrightii*. However, these effects did not match the predictions for negative frequency‐dependent selection. While near‐neighbor associational effects do not appear to underlie the maintenance of the *D. wrightii* trichome dimorphism, it is entirely possible for undetected effects to be at play. For example, herbivore populations often vary over the growing season of their hosts, and our visitation times (late April/early May for the spring 2018 visitation; late July/ early August for both summer measurements) may not correspond to the period in which these effects occur. It is also possible—given the degree to which herbivory varies from population to population (Goldberg et al., 2020)—that associational effects were occurring within some, but not all, of the populations we visited. In addition, the 1m scale at which we looked for associational effects may not match the scale upon which the processes underlying negative frequency‐dependent selection are playing out and previous studies have noted the importance of scale when assessing the roles of associational effects (Underwood et al., 2014). Nevertheless, we showed that near‐neighbor associational effects occur in the *D. wrightii* system, laying the groundwork for future studies into the maintenance of the balanced trichome dimorphism in California populations of *D. wrightii*.

## CONFLICT OF INTEREST

The authors declare no conflicts of interest.

## AUTHOR CONTRIBUTIONS

**Jay K. Goldberg:** Conceptualization (lead); Data curation (lead); Formal analysis (lead); Funding acquisition (lead); Investigation (lead); Methodology (lead); Project administration (lead). **Sonya R. Sternlieb:** Investigation (supporting); Project administration (supporting). **Genevieve Pintel:** Investigation (supporting); Project administration (supporting). **Lynda F. Delph:** Supervision (supporting); Writing‐original draft (supporting).

## Data Availability

The raw data and code associated with this manuscript are available at https://doi.org/10.5061/dryad.63xsj3v1v

## References

[ece37454-bib-0001] Baldwin, I. T. , Halitschke, R. , Paschold, A. , von Dahl, C. C. , & Preston, C. A. (2006). Volatile signaling in plant‐plant interactions: “Talking Trees” in the genomics era. Science, 311, 812–815. 10.1126/science.1118446 16469918

[ece37454-bib-0002] Baldwin, I. T. , & Schultz, J. C. (1983). Rapid changes in tree leaf chemistry induced by damage: Evidence for communication between plants. Science, 221, 277–279. 10.1126/science.221.4607.277 17815197

[ece37454-bib-0003] Barbosa, P. , Hines, J. , Kaplan, I. , Martinson, H. , Szczepaniec, A. , & Szendrei, Z. (2009). Associational resistance and associational susceptibility: Having right or wrong neighbors. Annual Review of Ecology, Evolution, and Systematics, 40, 1–20. 10.1146/annurev.ecolsys.110308.120242

[ece37454-bib-0004] Bonsall, M. B. , & Hassel, M. P. (1997). Apparent competition structures ecological assemblages. Nature, 388, 371–373. 10.1038/41084

[ece37454-bib-0005] Castagneyrol, B. , Giffard, B. , Péré, C. , & Jactel, H. (2013). Plant apparency, an overlooked driver of associational resistance to insect herbivory. Journal of Ecology, 101, 418–429.

[ece37454-bib-0006] Elle, E. , & Hare, J. D. (2000). No benefit of glandular trichome production in natural populations of Datura wrightii? Oecologia, 123, 57–65. 10.1007/s004420050989 28308744

[ece37454-bib-0007] Elle, E. , van Dam, N. M. , & Hare, J. D. (1999). Cost of glandular trichomes, a “resistance” character in *Datura wrightii* regel (Solanaceae). Evolution, 53, 22–35. 28565189 10.1111/j.1558-5646.1999.tb05330.x

[ece37454-bib-0008] Gassmann, A. J. , & Hare, J. D. (2005). Indirect cost of a defensive trait: Variation in trichome type affects the natural enemies of herbivorous insects on *Datura wrightii* . Oecologia, 144, 62–71. 10.1007/s00442-005-0038-z 15800744

[ece37454-bib-0009] Goldberg, J. K. , Lively, C. M. , Sternlieb, S. R. , Pintel, G. , Hare, J. D. , Morrissey, M. B. , & Delph, L. F. (2020). Herbivore‐mediated negative frequency‐dependent selection underlies a trichome dimorphism in nature. Evolution Letters, 4, 83–90. 10.1002/evl3.157 32055414 PMC7006469

[ece37454-bib-0010] Hambäck, P. A. , Inouye, B. D. , Andersson, P. , & Underwood, N. (2014). Effects of plant neighborhoods on plant–herbivore interactions: Resource dilution and associational effects. Ecology, 95, 1370–1383. 10.1890/13-0793.1 25000768

[ece37454-bib-0011] Hare, J. D. (2007). Variation in herbivore and methyl jasmonate‐induced volatiles among genetic lines of *Datura wrightii* . Journal of Chemical Ecology, 33, 2028–2043. 10.1007/s10886-007-9375-1 17960462

[ece37454-bib-0012] Hare, J. D. , & Elle, E. (2001). Geographic variation in the frequencies of trichome phenotypes of *Datura wrightii* and correlation with annual water deficit. Madroño, 48, 33–37.

[ece37454-bib-0013] Hare, J. D. , & Elle, E. (2002). Variable impact of diverse insect herbivores on dimorphic *Datura Wrightii* . Ecology, 83, 2711–2720.

[ece37454-bib-0014] Hare, J. D. , & Sun, J. J. (2011). Production of induced volatiles by *Datura wrightii* in response to damage by insects: Effect of herbivore species and time. Journal of Chemical Ecology, 37, 751–764. 10.1007/s10886-011-9985-5 21691808

[ece37454-bib-0015] Hay, M. E. (1986). Associational plant defenses and the maintenance of species diversity: Turning competitors into accomplices. American Naturalist, 128, 617–641. 10.1086/284593

[ece37454-bib-0016] Holt, R. D. , & Bonsall, M. B. (2017). Apparent competition. Annual Review of Ecology, Evolution, and Systematics, 48, 447–471. 10.1146/annurev-ecolsys-110316-022628

[ece37454-bib-0017] Joo, Y. , Schuman, M. C. , Goldberg, J. K. , Kim, S. , Yon, F. , Brütting, C. , & Baldwin, I. T. (2017). Herbivore‐induced volatile blends with both “fast” and “slow” components provide robust indirect defense in nature. Functional Ecology, 32, 136–149.

[ece37454-bib-0018] Kalske, A. , Shiojiri, K. , Uesugi, A. , Sakata, Y. , Morrell, K. , & Kessler, A. (2019). Insect herbivory selects for volatile‐mediated plant‐plant communication. Current Biology, 29, 3128–3133. 10.1016/j.cub.2019.08.011 31522939

[ece37454-bib-0019] Kogan, M. , & Goeden, R. D. (1970). The host‐plant range of *Lema trilineata daturaphila* (Coleoptera: Chrysomelidae). Annals of the Entomological Society of America, 63, 1175–1180.

[ece37454-bib-0020] Latif, S. , Chiapusio, G. , & Weston, L. A. (2017). Chapter Two – Allelopathy and the role of allelochemicals in plant defense. In G. Becard (Ed.), Advances in botanical research, how plants communicate with their biotic environment (pp. 19–54). Academic Press.

[ece37454-bib-0021] Lee, G. , Joo, Y. , Diezel, C. , Lee, E. J. , Baldwin, I. T. , & Kim, S.‐G. (2016). *Trichobaris* weevils distinguish amongst toxic host plants by sensing volatiles that do not affect larval performance. Molecular Ecology, 25, 3509–3519. 27146082 10.1111/mec.13686

[ece37454-bib-0022] Reinecke, J. P. , Buckner, J. S. , & Grugel, S. R. (1980). Life cycle of laboratory‐reared tobacco hornworms, *Manduca sexta*, a study of development and behavior, using time‐lapse cinematography. Biological Bulletin, 158, 129–140.

[ece37454-bib-0023] Rose, C. E. , Martin, S. W. , Wannemuehler, K. A. , & Plikaytis, B. D. (2006). On the use of zero‐inflated and hurdle models of modeling vaccine adverse event count data. Journal of Biopharmaceutical Statistics, 4, 463–481. 10.1080/10543400600719384 16892908

[ece37454-bib-0024] Sato, Y. , Kawagoe, T. , Sawada, Y. , Hirai, M. Y. , & Kudoh, H. (2014). Frequency‐dependent herbivory by a leaf beetle, *Phaedon brassicae*, on hairy and glabrous plants of *Arabidopsis halleri* subsp. gemmifera. Evolutionary Ecology, 28, 545–559.

[ece37454-bib-0026] Sato, Y. , & Kudoh, H. (2016). Associational effects against a leaf beetle mediate a minority advantage in defense and growth between hairy and glabrous plants. Evolutionary Ecology, 30, 137–154. 10.1007/s10682-015-9809-0

[ece37454-bib-0027] Underwood, N. , Inouye, B. D. , & Hambäck, P. A. (2014). A conceptual framework for associational effects: When do neighbors matter and how would we know? The Quarterly Review of Biology, 89, 1–19. 10.1086/674991 24672901

[ece37454-bib-0028] van Dam, N. M. , Hare, J. D. , & Elle, E. (1999). Inheritance and distribution of trichome phenotypes in *Datura wrightii* . Journal of Heredity, 90, 220–227. 10.1093/jhered/90.1.220

[ece37454-bib-0029] Vasconellos‐Neto, J. , Romero, G. Q. , Santos, A. J. , & Dippenaar‐Schoeman, A. S. (2007). Associations of spiders of the genus Peucetia (Oxyopidae) with plants bearing glandular hairs. Biotropica, 39, 221–226. 10.1111/j.1744-7429.2006.00250.x

[ece37454-bib-0030] Westdal, P. H. , & Romanow, W. (1972). Observations on the biology of the flea beetle, *Phyllotreta cruciferae* (Coleoptera: Chrysomelidae). Manitoba Entomology, 6, 35–45.

[ece37454-bib-0031] White, J. A. , & Whitham, T. G. (2000). Associational susceptibility of cottonwood to a box elder herbivore. Ecology, 81, 1795–1803.

[ece37454-bib-0032] Zakir, A. , Sadek, M. M. , Bengtsson, M. , Hansson, B. S. , Witzgall, P. , & Anderson, P. (2013). Herbivore‐induced plant volatiles provide associational resistance against an ovipositing herbivore. Journal of Ecology, 101, 410–417. 10.1111/1365-2745.12041

